# Occupational heat stress among female agricultural workers in the Jordan Valley, Palestine

**DOI:** 10.1093/annweh/wxag048

**Published:** 2026-06-22

**Authors:** Maysaa Nemer, Suzan Mitwalli, Hanin Basha

**Affiliations:** Institute of Community and Public Health, Birzeit University, PO Box 14, P627 Birzeit, West Bank, Palestine; Institute of Community and Public Health, Birzeit University, PO Box 14, P627 Birzeit, West Bank, Palestine; Institute of Community and Public Health, Birzeit University, PO Box 14, P627 Birzeit, West Bank, Palestine

**Keywords:** occupational heat stress, female agricultural workers, Jordan Valley, Palestine, wet bulb globe temperature, heat-related illness, climate change

## Abstract

**Introduction:**

Heat stress and extreme weather events are increasingly recognized as significant consequences of climate change, disproportionately affecting vulnerable populations, whose exposure and adaptive capacity are shaped by social, political, and economic conditions. In the context of occupational health, workers engaged in outdoor labor are among the most exposed groups, with agricultural workers facing particularly high risks due to prolonged exposure to high temperatures and extreme weather conditions. In politically constrained and resource-limited settings such as the Jordan Valley, Palestine, female agricultural workers face intersecting environmental, gendered, and structural vulnerabilities that may intensify heat-related health risks.

**Objective:**

This study examined heat exposure and its associated health effects among female agricultural workers in the Jordan Valley, West Bank, Palestine.

**Methods:**

A cross-sectional study was conducted between February and August 2020 in 8 agricultural communities. A total of 356 women aged 18 yr and above participated in structured interviews assessing socio-demographics, work conditions, heat exposure, hydration, and heat-related symptoms, including kidney disease. Meteorological data for June to August 2019 were obtained from 3 regional weather stations and used to calculate the wet bulb globe temperature (WBGT) levels during peak agricultural months. Blood and urine tests were done among 35 women.

**Results:**

Participants had a mean age of 38.9 yr and an average of 16.1 yr of agricultural work experience. Most women worked ≥6 d/wk, and 59.5% reported performing vigorous work. The majority (73.8%) worked mainly outdoors or in greenhouses, with 94.4% reporting summer heat as a problem. Peak WBGT values reached 35.6 °C in Jericho at midday. Common symptoms included tiredness (43.5%), dizziness (44.6%), and severe headache (45.6%), significantly associated with lack of access to rest areas, toilets, or drinking water (all *P* < 0.001). Multivariate analysis showed that women performing vigorous work had increased odds of excessive sweating, thirst, and fatigue (*P* < 0.001). Kidney stone prevalence was also significantly associated with heavy work, water scarcity, and lack of toilet access. Statistically significant WBGT differences were found between women reporting and not reporting heat-related symptoms, with the highest difference observed for tiredness at 0.812 °C (*P* < 0.05).

**Conclusion:**

Female agricultural workers in the Jordan Valley experience substantial occupational heat stress shaped not only by climatic exposure but also by gendered labor precarity, inadequate workplace infrastructure, and restricted adaptive capacity under political constraints. By situating heat stress within structural vulnerability, this study contributes to global discussions on climate-linked occupational health and highlights the need for gender-sensitive, context-specific interventions in climate-stressed and politically marginalized agricultural regions.

What's Important About This Paper This study represents the first comprehensive assessment of workplace heat stress levels among Palestinian female agricultural workers. It reveals the difficult working conditions, prolonged exposure to high temperatures, and the lack of essential workplace facilities that threaten women's health. The findings highlight an overlooked working population that is doubly marginalized, by gender and by the impacts of climate change. This research could help shape public health policies, work regulations, and climate adaptation initiatives in resource-limited agricultural settings.

## Introduction

Being exposed to extreme heat leads to increased heat storage in the body, causing a rise in body temperature and possibly leading to various illnesses such as heat stroke, rashes, cramps, and exhaustion, collectively known as heat stress (The National Institute for Occupational Safety and Health 2024; World Health Organization 2024). Heat stress is an emerging health risk and is exacerbated by global climate change, which is contributing to more frequent extreme weather events and heat waves (El Khayat et al. 2022). Direct sun exposure, high temperatures, and humidity increase the risk of heat stress symptoms, affecting vulnerable populations such as the elderly and people with chronic diseases, as well as outdoor workers (International Labour Office 2011; The National Institute for Occupational Safety and Health 2024; World Health Organization 2024). Among these, farmworkers are particularly at risk, especially when working in unsafe conditions with limited protective measures (El Khayat et al. 2022). Heat-related illnesses and symptoms as well as kidney disease have been identified as the most studied climate-linked health risks for farmworkers (El Khayat et al. 2022).

There are several methods for assessing occupational heat stress, which can be broadly divided into environmental indices and physiological measurements (Rachid and Qureshi 2023). Heat index, wet bulb globe temperature (WBGT), and universal thermal climate index are some examples for the environmental indices, while physiological measurements assess indicators like body temperature and heart and sweat rates (Feng et al. 2022). The WBGT is a widely accepted environmental index that incorporates air temperature, humidity, wind speed, and solar radiation to assess heat effect on the human body (Brimicombe et al. 2023). It has been applied widely in occupational heat exposure studies across different hot-climate regions. For example, in East Africa, heat levels were estimated through WBGT showing that farmers are facing heat stress, and their ability to work safely with good productivity is at risk (Yengoh and Ardö 2020). Similarly, a study in Saudi Arabia applied WBGT to assess heat exposure among construction workers (Al-Bouwarthan et al. 2019).

People living in poor, rural areas with limited resources and low-quality houses are more vulnerable to heat stress (World Health Organization 2024). In the West Bank, the Jordan Valley is known for its rich and fertile land, making it the most critical region for agricultural activity in Palestine (Melon 2018; Salem 2019). However, the Jordan Valley also records the highest temperatures in the West Bank (Richard and Issac 2012). Meteorological data from stations located in and around the Jordan Valley (Jenin, Jericho, and Nablus) show the extreme summer heat, as presented in a report by the Palestine Meteorological Department. In 2018, absolute maximum temperatures during June–August reached 44.8 °C in Jericho, 41.4 °C in Jenin, and 39 °C in Nablus (Palestinian Meteorological Authority 2018). Highest temperature, highest sunshine, and lowest rainfall were recorded at the Jericho station (Palestinian Central Bureau of Statistics 2025).

Furthermore, 90% to 95% of the Jordan Valley land falls under “Area C”, a designation under the Oslo Accords that places the area under full Israeli civil and military control (Melon 2018). Israeli practices in these areas aim to create extremely hard living conditions for Palestinians, to force them leave their home. These include illegal seizure of Palestinian land, exploitation of Palestinian natural resources, and very strict restrictions on construction, land and water access, and movement. Therefore, Palestinians in the Jordan Valley are not allowed to build houses, and any construction is subject to demolition (Melon 2018; Palestine Economic Policy Research Institute (MAS) et al. 2025). Households in the Jordan Valley face some of the most challenging living conditions in the West Bank with many experiencing low standards of living (Hammami 2019), lacking access to water networks, relying on tanked water (Abu Awwad 2016), and coping with unreliable or absent electricity (Jarrar 2018). Consequently, these predominantly farming communities face significant political, social, and economic challenges.

Women play a central role in agricultural production in the West Bank and Gaza, engaging in land preparation, planting, harvesting, food processing, packaging, animal husbandry, and marketing (Abu Awwad 2016; Salem 2019). Palestinian women farmers face a unique intersection of political, economic, and social challenges (Abu Awwad 2016). Approximately 90% of women in the informal workforce are employed in agriculture (Salem 2019), often in seasonal or temporary roles on family farms, where their labor is undervalued or treated as an extension of household duties (Food and Agricultural Office of the United Nations 2011; Abu Awwad 2016). Emerging scholarship emphasizes that environmental vulnerability is not solely a function of climatic exposure but is socially and politically produced through structural inequalities that constrain coping and adaptation capacity (Mason et al. 2012; Hamstead 2024). In contexts marked by restricted land use, limited water access, and constrained infrastructure development, these structural conditions may intensify occupational heat risk.

Despite the documented climatic extremes and socio-political constraints in the Jordan Valley, limited research has examined how environmental heat exposure interacts with gendered labor precarity and infrastructural deficits to affect women's health. Most occupational heat studies focus primarily on physiological exposure, with less attention to how political economy, governance, and access restrictions shape vulnerability. This study, therefore, aims to examine heat exposure and its associated health effects among female agricultural workers in the Jordan Valley, West Bank, situating occupational heat stress within its broader socio-political and gendered context.

## Methods

### Study design and sample

A cross-sectional study was conducted from February to August 2020, in 8 selected agriculture communities of the Jordan Valley, West Bank, Palestine. The study targeted all agricultural working women aged 18 yr and above who were residing in the Palestinian Jordan Valley area in 2020. According to the Office for the Coordination of Humanitarian Affairs classification (Office for the Coordination of Humanitarian Affairs occupied Palestinian territory 2012), the Jordan Valley area covers about 29 communities, most of which are small in population. According to the Palestinian Central Bureau of Statistics census of 2017 (Palestinian Central Bureau of Statistics 2018), the total number of working women in the Jordan Valley was about 2,594 women, who were working in agriculture and other sectors, but we expected that at least half of these women are working in agricultural work, given the nature of the area and the most common jobs available. According to these data, an estimated sample size of 356 women was obtained using the typical formula of sample size calculation (Cochran 1977). We used a 2-stage stratified cluster sampling, in which (i) we selected a probability proportional to the size of the sample of communities/localities within the Valley, covering the 3 main Governorates (Jericho, Jenin, and Nablus) and (ii) we selected a sample of female farmers working in each locality within the first stage (See Supplementary Material S1 for more details about the sampling methodology). This resulted in selecting a total of 356 farming women across 7 Jordan Valley communities, which are highlighted in the map shown in Supplementary Material S2.

### Study tools and data collection

The study utilized an interview-administered questionnaire, which included questions on demographic and socioeconomic conditions, working conditions, heat exposure at work, hydration status at work, and health effects (heat-related symptoms and kidney disease). Five locally trained female fieldworkers collected the data in the selected communities through home or farm visits to the participants and using an electronic version of the questionnaire, which was scripted in remote data collection software (Mobile Data Studios—MDS). Within each selected study community, data collectors walked through the community and approached potential participants randomly at their homes or workplaces, where they explained the purpose of the study and invited them to participate. Participants who declined were asked to provide their reason for refusal.

Weather data for the measured climatic parameters were obtained from the Palestinian Meteorological Department, which operates measurement stations in Jericho, Jenin, and Nablus. These stations cover the 7 Jordan Valley communities included in the study. The Palestinian Meteorological Department collects data using different manual and automatic equipment (Palestinian Meteorological Authority 2018). For this study, we requested data for the 3 mo that were considered by the study participants as the ones with the highest temperature of the past agricultural season: June, July, and August 2019. The data obtained include dry bulb temperature (°C), relative humidity (%), and wind speed (m/s), which were measured daily in each month, and at a 3-h interval, starting from midnight until 21:00. Due to the lack of data on solar radiation, WBGT was calculated using the standard formula for the shade/indoors (WBGT = 0.7XTnwb + 0.3XTg). Average, minimum, and maximum values were calculated for the times of the day when the farmers perform most of their workload: at 6:00, 9:00, 12:00, and 15:00. Then, the peak and mean WBGT values among the 4 timings were identified and used to determine the overall heat stress among workers, taking into consideration the work intensity during these timings.

Upon completion of the survey data collection, all participants were initially intended to undergo blood and urine testing. However, due to COVID-19-related restrictions that occurred during data collection, testing could only be conducted in 2 of the studied communities. Therefore, participants from these 2 communities who had completed the survey were invited to undergo blood and urine testing. Those who consented convened at the local council facilities on scheduled dates. The research team coordinated with a laboratory technician, who collected blood and urine samples. At the end of each day, the samples were transported to the medical laboratory for analysis to obtain serum creatinine (mg/dl) and urine specific gravity. A total of 34 blood and urine samples were collected. Urine specific gravity was used to assess hydration status, and the values were categorized as follows: ≤1.015, euhydrated (optimal hydration); 1.016 to 1.020, marginally adequate hydration; 1.021 to 1.025, hypohydrated; and 1.026 to 1.030, severely hypohydrated (Miller and Bates 2007).

### Data analysis

Statistical data analysis was performed using SPSS (Version 24, IBM) (IBM Corp 2024). Descriptive statistics were used to summarize and present the general characteristics of the women in the study communities, as well as the heat exposure and health effects variables. Means and standard deviations (SDs) were used to present continuous variables, and frequencies and percentages (*n*, %) were used to present categorical variables. Chi-square tests were conducted to examine the associations between dependent variables (reported heat-related health effects) and independent variables (demographic factors, working conditions, heat exposure, work intensity, and WBGT). If cell counts were less than 5, the Fisher's exact test was used instead. One-way ANOVA was used to compare the WBGT values between different work intensity categories, and an independent sample *t*-test was used to test the differences in mean WBGT values between farmers with and without symptoms.

Binary logistic regression was performed to identify predictors of heat-related health effects. Variables that showed significant differences in the bivariate analysis and other variables that showed a substantial but not significant difference and were also reported in previous studies as important predictors of heat-related health effects were included in the multivariate model. The results were reported as adjusted odds ratios (AORs) with 95% confidence intervals (CIs). Model fit was assessed using the Hosmer–Lemeshow test. A 2-tailed *P*-value <0.05 was considered statistically significant.

## Results

### General characteristics and living conditions

A total of 356 women participated in the study, with a non-response rate of 2.2% (8 workers). The mean age of the participants was 38.9 ± 10.3 yr, with a range of 18 to 50 yr. Most women (43.5%) had only primary education, and 62.9% were married. Regarding women's income from working in agriculture, 17.1% of the sample had monthly income with an average of 1,693 ± 525 shekels (467.2 USD), while 30.9% had daily salary with an average of 80.6 ± 16 shekels (22.2 USD). 37% of the women reported working with the family without payment, and 8.7% of them stated that they own the farm they work on. Table 1 shows the general characteristics of the sample.

**Table 1:** General characteristics of the participants.

Characteristics		Total sample *n* = 356		Sub-sample *n* = 34
		Mean (SD)		Mean (SD)
Age in years		38.9 (10.3)		41.4 (8.9)
Number of family members		6.2 (2.5)		6.7 (2.6)
Number of rooms		3.3 (1.1)		3.18 (0.9)

Income	*N* (%)	Mean (SD)	*N* (%)	Mean (SD)
Daily salary	110 (30.9)	80.59 (15.9)	11 (32.4)	58.64 (11.9)
Weekly salary	10 (3.1)	363.5 (158.9)	4 (11.8)	458.3 (94.6)
Monthly salary	62 (17.1)	1,693.6 (525.4)	4 (11.8)	1,975 (377.5)
Unpaid work	132 (37.1)	…	15 (44.1)	…
Farm owners	31 (8.7)	…	0	…
Profit (last 3 seasons, 2019)	44 (12.4)	23,227.3 (36,390.6)	2 (5.9)	7,500 (3,535.5)

Characteristics		*N* (%)		*N* (%)
Educational attainment				
Illiterate		26 (7.3)		4 (11.8)
Primary education		155 (43.5)		17 (50)
Secondary education		134 (37.6)		11 (32.3)
Higher than secondary		41 (11.5)		2 (5.9)
Marital status				
Single		104 (29.2)		2 (5.9)
Married		224 (62.9)		29 (85.3)
Divorced/widowed		28 (7.8)		3 (8.8)

### Working conditions

On average, participants had 16.1 ± 12.3 yr of farming experience, with almost half of them reporting more than 15 yr of work. Most of the women (45%) worked on family farms, where they contributed to their family's business, while others were employed in Palestinian farms owned by non-family members or in Israeli settlements. A smaller proportion (8.4%) reported owning and working on their private farms. The majority worked throughout most of the year, more than 5 d a week, and for 8 h or more per day usually with 1 short break. Almost two-thirds reported doing work that involved vigorous physical activity. In terms of agricultural tasks, they performed the tasks of land preparation, harvesting, weeding, and planting.

In terms of workplace conditions, findings highlight the limited availability of basic facilities for many participants. More than half of the women reported no access to toilets, nor places for resting, eating, drinking, and hand washing. Two-thirds had access to drinking water, and about one-third reported the presence of first aid or emergency materials. Of those who needed to go to a toilet during work, 29.7% reported waiting until they go home, while 22.1% used actual bathrooms located near the farm or relieved themselves in unused hidden buildings. Nearly half had to hold themselves back from not going to the toilet for longer periods than they could tolerate. Table 2 describes the working conditions of the participants.

**Table 2:** Working conditions of the participants (*n* = 356).

	*n*	%
**Working years in agriculture**		
0 to 5 yr	104	29.2
6 to 15 yr	96	27.0
16 to 25 yr	69	19.4
More than 25 yr	87	24.4
**Location of agricultural work**		
Private farm	30	8.4
Family farm	160	44.9
Palestinian farm	90	25.3
A farm in the Israeli settlements or subordinate to the settlements	76	21.3
**Working previously on other farms**		
Yes	144	59.6
No	212	40.4
**Working in any other job**		
Yes	47	13.2
No	309	86.8
**Number of months per year in agricultural work**		
5 or less mo	102	28.7
6 to 8 mo	83	23.3
9 to 12 mo	171	48.0
**Working days per week during the season**		
5 or less days	114	32
6 d	114	32
7 d	128	36
**Working hours per day during the season**		
Less than 8 h	198	55.6
8 h	110	30.9
More than 8 h	48	13.5
**Taking breaks during the day**		
Never	25	7
One break	249	70
Two or more breaks	82	23
**Work involving vigorous physical activity**		
Yes	246	69.1
No	110	30.9
**Workplace conditions**	***n***	**%**
**Workplace has a toilet**		
Yes	163	45.8
No	193	54.2
**Access to drinking water in the workplace**		
Yes	241	67.7
No	115	32.3
**Workplace has a location for resting, eating, drinking, and hand washing**		
Yes	173	48.6
No	183	51.4
**Workplace has first aid/emergency materials**		
Yes	109	30.7
No	221	62.3
**Agricultural tasks performed**		
Land preparation	98	27.5
Planting seeds and seedlings	248	69.7
Weeding	255	71.6
Harvesting	283	79.5

### Heat exposure

Most participants reported spending the majority of their work time outdoors or in the greenhouse, while only 26.1% reported spending their time in a building with a roof. A small percentage of women reported feeling comfortable with the ambient temperature while working in the summer season, while most women felt comfortable in spring, winter, and autumn. June, July, and August 2019 were identified as the months with the highest temperatures during the past agricultural season and were the focus of measured and reported heat exposure levels. Table 3 shows the calculated WBGT in the 3 governorates where the agricultural communities are located, and the charts in Supplementary Material S3 show the peak value of WBGT change over time during the workday for the 3 locations and during these months. The time for the peak value of WBGT was 12:00 PM for Jericho in both June and July, with WBGT peak values of 35.6 and 34.8, respectively. The same time was also reported for Jenin and Nablus in August, with peak WBGT values of 34.6 and 32.9, respectively. The second recorded time for the peak value of WBGT was 9:00 AM for Jenin in June (31.6) and July (34.5), and in July for Nablus with a peak WBGT of 32.6.

**Table 3:** Wet bulb globe temperature (WBGT) values in studied locations during June, July, and August 2019.

Date	Location	WBGT (°C)			
		Average (SD)	Minimum (at 6:00 AM)	Peak value	Time of peak value
June 2019	Jericho	25.6 (2.2)	16.9	35.6	12:00 PM
	Jenin	24.2 (1.3)	14.1	31.6	9:00 AM
	Nablus	22.4 (1.5)	6.73	32.5	12:00 PM
July 2019	Jericho	26.3 (2.0)	19.0	34.8	12:00 PM
	Jenin	25.2 (1.3)	17.3	34.5	9:00 AM
	Nablus	23.9 (1.6)	10.9	32.6	9:00 AM
August 2019	Jericho	26.9 (1.9)	15.3	34.3	3:00 PM
	Jenin	25.8 (1.1)	18.7	34.6	12:00 PM
	Nablus	24.0 (1.3)	28.7	32.9	12:00 PM

During these 3 mo, harvesting was the most commonly reported task (61%), followed by weeding (41.1%), planting seeds (32.7%), and land preparation (23.9%). Participants estimated both the amount of work defined as the number of days worked per month and the intensity of work defined as perceived effort required to perform agricultural tasks. Around one-third of the participants rated their amount and intensity of work to be 25% or less, while 39% rated the amount to be 75% or more, and 26.1% rated the intensity to be 75% or more. Calculated work intensity among the participants included light work, with 29.5% of the participants, moderate work, with 11% of them, and heavy work, which was identified among 59.5% of the participants (Table 4).

**Table 4:** Work tasks, amount, and intensity of work during the months of highest temperatures (June, July, August 2019).

	*n*	%
**Work tasks during the months of highest temperatures**		
Land preparation	85	23.9
Planting seeds and seedlings	116	32.7
Weeding	144	41.1
Harvesting	217	61.0
Packaging and loading boxes	49	13.8
**Rated amount of work**		
25% or less (worked for at most 7 d each month)	119	33.4
50% (worked 10 to 14 d each month)	98	27.5
75% or more (worked 15 to 21 d each month)	139	39
**Rated intensity of work**		
25% or less (never or rarely performed tasks that required considerable effort)	130	36.5
50% (performed tasks that required considerable effort, but not often)	133	37.4
75% or more (often performed tasks that required considerable effort)	93	26.1
**Work intensity**		
Light work	105	29.5
Moderate work	39	11
Heavy work	212	59.5

Table 5 summarizes the association between the 3 groups of work intensity and the difference in WBGT average and peak values. The WBGT threshold values were retrieved from the Occupational Safety and Health Administration (Occupational Health and Safety) and the National Institute for Occupational Safety and Health (The National Institute for Occupational Safety and Health 2016). The table shows that the peak WBGT values are higher than the threshold values for all work intensities. The results also show a statistically significant association between July and August 2019 peak WBGT for the group with light work, with peak WBGT values of 34.53 and 34.33, respectively. Additionally, the association was significant for the June 2019 peak value for the group with heavy work (34.70).

**Table 5:** Relationship between work intensity and WBGT (*n* = 356).

Work intensity	Average WBGT^a^ (°C)			Peak WBGT^a^ (°C)			WBGT threshold (°C)
	June 2019	July 2019	August 2019	June 2019	July 2019	August 2019	
Light work (*n* = 105)	24.75 (0.93)	25.67 (0.70)	26.18 (0.79)	33.56 (1.96)	34.53 (0.55)*	34.33 (0.41)*	30
Moderate work (*n* = 39)	24.65 (1.09)	25.58 (0.83)	26.07 (0.95)	33.62 (1.93)	34.41 (0.73)	34.22 (0.53)	27.8
Heavy work (*n* = 212)	24.79 (1.36)	25.68 (1.04)	26.15 (1.21)	34.70 (1.74)*	34.28 (0.97)	33.95 (0.60)	26.1

^a^Mean (SD).

^*^ *P* < 0.05.

ANOVA was used to assess the difference in WBGT peak values between the 3 groups of work intensity.

### Heat-related health effects

When asking respondents whether they had experienced any symptoms related to heat stress at work during the last 12 mo, the most frequently reported symptom was feeling tired and weak, affecting around 86.5% of respondents. The second most frequent symptom was severe headache, with a percentage of 57.9%, followed by excessive sweating, excessive thirst, and muscle/heat cramps, and dizziness. In terms of kidney disease, around 10% reported ever having kidney stones, who all reported being treated, while 70.8% reported having pain in their lower back (Table 6).

**Table 6:** Heat-related symptoms reported by the participants (*n* = 356).

Symptom	*n*	%
Tiredness/weakness	308	86.5
Severe headache due to heat	206	57.9
Excessive sweating	174	48.9
Excessive thirst	48	48
Muscle/heat cramps	133	37.4
Dizziness due to heat	121	34
Skin rash due to heat exposure	44	12.4
Nausea and vomiting	36	10.1
Heat stroke	24	6.7
Fainting due to heat	15	4.2

### Bivariate analysis

We conducted a bivariate analysis using the chi-square test to examine the associations between the most reported heat-related health effects and workplace determinants. Excessive thirst, severe headache, dizziness due to heat, tiredness/weakness, kidney stones, and pain in the lower back were significantly associated with most of the determinants, with the significant associations presented in Supplementary Material S4. Specifically, working in the greenhouse, the absence of a place for eating, drinking, and resting in the workplace, inaccessibility to a toilet in the workplace, lack of access to drinking water, working less than 8 h per day, and working 7 d/wk are significantly associated with most reported heat-related health effects. Other determinants significantly associated with some health effects were the governorate where the participants live, age group, intensity of work, years of work, and number of working months per year.

In terms of the association between reported symptoms and WBGT values, the results showed a statistically significant mean difference of the average WBGT and peak WBGT of the 3 mo (June, July, and August) of 2019 for some heat-related health effects. Specifically, there was a significant mean difference in WBGT between women who reported muscle/heat cramps and who didn’t, with the largest mean difference being 0.313 for the average WBGT of June 2019. For the excessive thirst symptom, the highest mean difference was between those who reported excessive thirst and those who didn’t (0.398) for the WBGT peak value of June 2019. Likewise, the highest mean difference between those who reported feeling tired and those who didn’t was 0.812 for the WBGT peak value of June. The highest significant mean difference was 0.371 of the average WBGT of June 2019 between women with kidney stones and those without.

### Sub-sample analysis

In addition to survey data and environmental heat exposure measurements, biological samples were collected from a subsample of participants to assess hydration status and potential renal stress associated with occupational heat exposure. Blood and urine analyses were conducted for 35 women.

The average serum creatinine for the sub-sample of 34 workers was 0.76 mg/dl (SD = 0.068), with a range of 0.58 −0.88. The average urine specific gravity was 1.024 (SD = 0.0057), with a range of 1.01 to 1.033. Among the 34 participants, 11.8% (*n* = 4) were classified as optimally hydrated, and 14.7% (*n* = 5) had marginally adequate hydration. Hypohydration was observed in 35.3% of participants (*n* = 12), and the largest proportion, 38.2% (*n* = 13), was severely hypohydrated. The analysis showed that there is a statistically significant association between those with both hypohydration and dehydration test results and working in the field (*P* < 0.001). Doing heavy work was also significantly associated with dehydration for these women (*P* < 0.05). The results also showed an association between doing vigorous work tasks and excessive sweating (*P* < 0.05). Moreover, the average and peak WBGT in the months of June, July, and August 2019 were all significantly associated with the group of women who were marginally or severely dehydrated (*P* < 0.05).

### Multivariate analysis

We conducted a multivariate analysis for the determinants significantly associated with the participants’ heat-related symptoms, adjusting for age. Table 7 summarizes the multivariate analysis. Women who reported doing vigorous work were more likely to have the symptoms of excessive sweating and excessive thirst (*P* < 0.001), and tiredness/weakness (*P* < 0.05). Likewise, women with moderate work intensity compared with those of light work were more likely to have excessive sweating (*P* < 0.001) and thirst (*P* < 0.05), and severe headache (*P* < 0.05). Women who reported a lack of access to drinking water were more likely to report excessive thirst and severe headache, with *P* < 0.05 and *P* < 0.001, respectively. Working in the field was also associated with severe headache, with women who reported working in the field were more likely to report severe headache (*P* < 0.001). For muscle/heat cramps symptoms, women who worked for 6 to 15 yr and worked during WBGT peak value were more likely to develop these symptoms (*P* < 0.05). Additionally, women who worked during WBGT peak were more likely to have tiredness/weaknesses (*P* < 0.05).

**Table 7:** Predictors of heat-related symptoms among the participants (*n* = 356).

	Excessive sweating			Muscle/heat cramps			Excessive thirst			Tiredness/weakness			Severe headache due to heat		
	OR^a^	95% CI	*P*-value	OR^a^	95% CI	*P*-value	OR^a^	95% CI	*P*-value	OR^a^	95% CI	*P*-value	OR^a^	95% CI	*P*-value
**Years of work**															
0 to 5 yr	(ref)			(ref)			(ref)			(ref)			(ref)		
6 to 15 yr	1.38	0.60–3.17	0.44	0.36	0.15–0.87	**0**.**02**	0.75	0.32–1.75	0.51	0.61	0.17–2.13	0.44	0.43	0.18–1.04	0.066
16 to 25 yr	1.34	0.68–2.66	0.39	0.94	0.47–1.86	0.86	1.20	0.60–2.39	0.60	0.53	0.18–1.52	0.24	0.50	0.24–1.02	0.055
More than 25 yr	1.28	0.64–2.56	0.48	0.92	0.46–1.84	0.82	0.88	0.43–1.78	0.72	0.98	0.27–3.53	0.97	0.88	0.41–1.86	0.73
**Place of work**															
Indoors	(ref)			(ref)			(ref)			(ref)			(ref)		
Field	0.71	0.34–1.46	0.35	1.28	0.61–2.65	0.50	0.64	0.31–1.33	0.23	0.84	0.33–2.12	0.72	0.36	0.17–0.77	**0**.**008**
Greenhouse	0.84	0.50–1.41	0.51	1.13	0.67–1.92	0.63	1.09	0.64–1.85	0.74	1.78	0.67–4.71	0.24	1.48	0.84–2.60	0.16
**Intensity of work**															
Light	(ref)			(ref)			(ref)			(ref)			(ref)		
Moderate	2.64	1.50–4.65	**0**.**001**	0.65	0.37–1.13	0.12	2.11	1.19–3.73	**0**.**01**	1.33	0.58–3.01	0.49	0.48	0.27–0.86	**0**.**015**
Heavy	2.08	0.96–4.48	0.061	0.56	0.25–1.25	0.16	1.50	0.69–3.25	0.30	0.54	0.21–1.43	0.21	0.83	0.38–1.84	0.65
**Vigorous work tasks**	0.21	0.12–0.39	**<0**.**001**	1.0	0.58–1.73	0.99	0.20	0.11–0.38	**<0**.**001**	0.34	0.16–0.73	**0**.**006**	0.59	0.33–1.05	0.07
**Access to drinking water**	0.78	0.42–1.45	0.44	1.06	0.57–1.97	0.84	0.45	0.23–0.85	0.**015**	0.422	0.13–1.31	0.13	0.27	0.13–0.54	**<0**.**001**
**Access to a toilet**	1.31	0.70–2.45	0.38	0.90	0.47–1.69	0.74	0.89	0.47–1.68	0.73	2.28	0.82–6.27	0.11	0.74	0.38–1.44	0.38
**WBGT Peak**	0.81	0.60–1.08	0.15	1.49	1.10–2.02	**0**.**01**	1.27	0.94–1.71	0.11	0.51	0.27–0.92	**0**.**028**	0.81	0.59–1.11	0.18

^**a**^Adjusted for age.

Bold indicates significant values.

## Discussion

This study provides a detailed assessment of occupational heat exposure and its associated health effects among female agricultural workers in the Jordan Valley, West Bank, Palestine. The findings reveal that these women are engaged in labor-intensive agricultural work tasks under harsh environmental conditions, with limited access to basic needs or safety at the workplace.

### Poor working conditions and gendered vulnerability

The findings of this research align with previous research indicating that agricultural workers in low- and middle-income countries (LMICs) often endure physically demanding labor under extreme weather conditions, frequently with insufficient protection (Kjellstrom et al. 2009; Levi et al. 2018; Amoadu et al. 2023). The extended work periods under high heat conditions are known to elevate the risk of heat-related illnesses and chronic dehydration (Amoadu et al. 2023). Additionally, vigorous tasks such as harvesting, weeding, and planting increase the risk of heat-related health effects when performed under elevated temperatures (Wagoner et al. 2020; El Khayat et al. 2022).

The absence of basic workplace facilities reported by many participants reflects broader structural vulnerabilities associated with workers in rural areas, particularly among agricultural laborers, who often lack formal workplace infrastructure. This has been seen on the global level among rural workers, who frequently experience deficits in decent work, including limited access to safe working environments and basic workplace amenities (Garland 2022). Evidence from a systematic review highlights the need for strong regulatory protections to ensure adequate occupational health and safety conditions among agricultural workers (Tran et al. 2026). Poor working conditions, such as the absence of essential facilities, not only compromise dignity and comfort but also increase vulnerability to heat stress and related health complications (El Khayat et al. 2022). The need to delay toilet use and the lack of private, accessible facilities may further contribute to physical stress and urinary health issues, compounding the effects of heat exposure, particularly among women (Diverde 2013; Venugopal et al. 2016, 2019). Chronic heat exposure can lead to fatigue, dehydration, heat exhaustion, and even long-term cardiovascular and renal consequences, especially for workers in physically demanding jobs (Cheveldayoff et al. 2023). Moreover, heat stress disproportionately affects women in informal labor sectors, who often lack protective labor regulations, access to cooling interventions, or healthcare coverage (International Labour Organization 2024).

Regional and global studies highlight the disproportionate burden of climate-related occupational hazards borne by agricultural workers, especially women (Venugopal et al. 2019; Glazebrook et al. 2020; Pal et al. 2021; Venugopal et al. 2021). In similar contexts across the Global South, the intersection of gender, poverty, and informal employment magnifies vulnerability to environmental stressors (Ngcamu 2023). Therefore, our study emphasizes the need for targeted interventions to reduce occupational heat exposure, such as regulated rest breaks, provision of shade and hydration, and enforcement of safety standards, particularly in informal or family-run farm settings (Occupational Safety and Health Administration 2021; International Labour Organization 2024).

### Heat exposure and heat discomfort

In addition to long working hours and limited access to basic facilities, most women in this study reported spending the majority of their workday outdoors or inside greenhouses; environments known to exacerbate heat stress due to limited ventilation and high humidity (Kjellstrom et al. 2018). The high percentage of women reporting discomfort with ambient temperatures during the summer season and the significant amount of time spent in greenhouses suggest dangerous levels of occupational heat stress.

Greenhouses, although providing crop protection, frequently function as heat traps. In this study, over a third of the participants indicated that they spent the majority of their day in greenhouses, with over a fifth dedicating their entire time indoors. Enclosed environments markedly increase WBGT levels, exceeding acceptable limits for prolonged high-intensity work, especially when rest and water are insufficient (Schulte and Chun 2009; Occupational Safety and Health Administration 2021; International Labour Organization 2024). In accordance with this, nearly all participants recognized heat exposure in summer as a serious problem, with a small proportion reporting experiencing comfort during this season, providing clear indicators of heat discomfort and stress. These findings are consistent with the literature demonstrating that greenhouses and open fields, especially in arid climates like the Jordan Valley, amplify heat exposure risks (Jackson and Rosenberg 2010; Karakuş et al. 2024). The reported discomfort during summer and comfort in cooler seasons confirms the seasonal variation of heat stress exposure, a pattern also observed in other agricultural settings globally (International Labour Office 2019; Varghese et al. 2019; Matallah et al. 2022).

While reported comfort levels were considerably higher during the spring, winter, and autumn, the perceived heat burden during the summer months, especially June, July, and August, aligns with WBGT measurements taken in the field. More than half of the women experienced heat-related discomfort for 4 to 6 mo annually, reinforcing findings from climate-health literature that global warming is expanding seasonal heat exposure in vulnerable communities (Intergovernmental Panel on Climate Change 2022).

### Health effects of heat stress

Compared with agricultural workers in other hot-climate regions, the prevalence of heat-related symptoms in this cohort appears notably high. Studies among sugarcane workers in Central America report fatigue prevalence ranging from 40% to 60% (Crowe et al. 2015), whereas 86.5% of women in our sample reported tiredness or weakness. This comparison suggests that the Jordan Valley cohort may experience a compounded vulnerability arising from climatic exposure combined with political constraints and infrastructural challenges, with the absence of sanitation, rest areas, and water access. Tiredness, weakness, severe headache, excessive sweating, thirst, muscle cramps, and dizziness are clear indicators of both acute and cumulative heat stress, particularly among workers engaged in continuous, physically demanding jobs under high-temperature conditions (Jacklitsch et al. 2016). The international literature identifies agricultural workers as among the most vulnerable groups to heat-related illness, particularly in low-resource settings with limited occupational health protections (Ireland et al. 2023; Faria et al. 2024; Junkesa et al. 2024). Women, in particular, face compounded risks due to overlapping vulnerabilities, including informal work arrangements, gender norms limiting access to healthcare, and the physiological impacts of heat on hydration and reproductive health (Kuehn and McCormick 2017; International Labour Office 2019).

Additionally, inadequate access to drinking water and toilets not only heightens the risk of acute heat illness but may also contribute to chronic issues such as dehydration-related kidney stones, which is supported by epidemiological studies linking occupational heat exposure to renal dysfunction (Glaser et al. 2016; Wesseling et al. 2016; Chapman et al. 2021).

### WBGT and physiological burden

When WBGT values were analyzed in relation to work intensity, a statistically significant difference was found: workers performing moderate to heavy labor experienced greater heat stress than those engaged in light tasks. This finding aligns with existing literature emphasizing the combined effects of environmental heat and metabolic workload in determining occupational heat strain (Flouris et al. 2018; Ioannou et al. 2022). Notably, the peak WBGT values in Jericho exceeded the recommended occupational exposure limits for continuous work under moderate and heavy exertion levels (Occupational Health and Safety; Occupational Safety and Health Administration 2017), underscoring the urgent need for effective protective strategies, including rest breaks, hydration, and heat adaptation policies in outdoor agricultural settings.

The WBGT analysis provides empirical support for the subjective heat-related symptoms reported. Significant differences in mean WBGT values between those with and without symptoms such as muscle cramps, excessive thirst, and fatigue substantiate the role of high environmental heat exposure in driving physiological stress. Previous studies validate WBGT as a strong predictor of occupational heat risk in both indoor and outdoor agricultural work environments (Venugopal et al. 2021; Brimicombe et al. 2023).

Our findings underscore that vulnerability to occupational heat stress extends beyond physiological exposure, reflecting deeper socio-political and gendered dimensions. Female agricultural workers in the Jordan Valley face heightened risks not only due to task-related heat exposure but also because of structural conditions, including informal labor arrangements, limited occupational health protections, and constrained adaptive options under political restrictions. This perspective aligns with scholarship that locates environmental vulnerability as socially and politically constructed rather than solely biophysical. For example, Mason, et al. (2012) show how occupation practices intensify climate risks in Palestine by restricting coping and adaptation capacity (Mason et al. 2012). Similarly, Hamstead (2024) introduces the idea of “thermal insecurity,” arguing that heat (and cold) threats are forms of structural violence intimately bound up with housing, energy access, and social determinants (Hamstead 2024). Robinson (2025) further highlights that heat is not just a physical variable (temperature) but also a socio-political phenomenon that is produced and produces inequality, power, infrastructure, culture, and governance (Robinson 2025). In this sense, our study extends existing knowledge by situating heat stress within a context of gendered precarity, contested political power, and restrictive access, highlighting the intersection of several vulnerabilities that influence health risks. Such an approach resonates with the concept of “thermal violence,” whereby extreme heat disproportionately harms marginalized groups whose ability to adapt is hindered by structural inequalities (Anwar 2023).

### Implications for policy and practice

The findings call for urgent occupational health interventions for this group of workers, including redesigning work-rest schedules based on real-time WBGT readings, installing shaded rest areas with ventilation, and improving access to drinking water. Furthermore, health surveillance systems should track heat-related symptoms and outcomes in agricultural populations, with disaggregated data by gender to better inform policy responses. Worker awareness and early warning systems for extreme heat events are also important to prevent adverse health outcomes. Relevant stakeholders should also consider integrating greenhouse thermal safety standards and heat-mitigating agricultural practices to protect workers in these enclosed environments.

Gender-specific occupational health interventions are also essential. The invisibility of female agricultural workers in health policy and occupational safety discourses exacerbates their vulnerability to climate-related hazards. Addressing occupational heat stress for this population requires not only infrastructural improvements but also empowerment strategies that include women in decision-making about workplace health and safety.

The findings suggest a need for policy initiatives that address both the physical and social vulnerabilities of women in agriculture. One such initiative could involve the implementation of comprehensive workplace heat stress management strategies, including regular monitoring of environmental conditions (eg, WBGT) and the provision of adequate rest periods, drinking water, and access to toilets. Furthermore, public health campaigns aimed at raising awareness about the dangers of heat stress and the importance of hydration and rest could help empower workers to take proactive steps to protect their health.

### Recommendations for future research

Given the strong associations between heat exposure, workplace infrastructure, and health effects, future research should focus on longitudinal studies that examine the long-term impact of chronic heat exposure on the health of agricultural workers. Studies that assess the effectiveness of intervention programs, such as the introduction of shade structures, improved water access, and regulated working hours, would provide valuable insights into strategies for mitigating heat stress in agricultural settings.

Additionally, more research is needed to explore the gendered dimensions of agricultural work and how heat stress disproportionately affects female workers. Exploring the intersection of socioeconomic status, educational background, and access to resources could offer deeper insights into the factors contributing to heat-related health disparities.

### Strengths and limitations of the study

A strength of this study is its focus on female agricultural workers, a population that remains underrepresented in occupational heat exposure research despite their central role in agricultural labor in many regions. Another strength lies in the integration of multiple sources of evidence to assess occupational heat stress, including self-reported work conditions and symptoms, physiological measurements, and environmental heat exposure estimated using meteorological data from official measurement stations. The use of WBGT calculations based on regional meteorological data provided an empirical environmental indicator to contextualize the working conditions experienced by participants in the Jordan Valley. This study was conducted in the Jordan Valley, the West Bank, a unique context where extreme climatic conditions intersect with social, economic, and political challenges that may shape workers' exposure and adaptive capacity. Examining occupational heat stress within this context contributes to expanding the evidence base on climate-related occupational health risks in under-studied settings.

However, 2 key limitations of our WBGT estimation must be acknowledged. First, we did not measure WBGT values at a specific point in time when conducting physiological measurements. Instead, we used the average WBGT values of the hottest months (June to August 2019) as a proxy for the heat exposure conditions typically experienced by participants. Second, the WBGT value calculations relied on standard meteorological data on air temperature, relative humidity, and wind speed, without direct inclusion of solar radiation, a critical factor influencing outdoor heat exposure. WBGT is ideally measured on-site using specialized instrumentation that includes a globe thermometer to account for radiant heat. When solar radiation is not directly measured, as in our study, the resulting WBGT may underestimate the actual heat load experienced by outdoor workers, particularly under direct sunlight (Mirzabeigi et al. 2021). Consequently, the true occupational heat stress in settings like Jericho may be even greater than indicated by our estimates. Future assessments should strive to incorporate solar radiation measurements or apply correction factors to provide a more precise assessment of occupational heat exposure for outdoor workers.

An additional limitation relates to the temporal and spatial resolution of the heat exposure assessment compared with the self-reported health outcomes. WBGT values were calculated using average meteorological data for the hottest months (June to August 2019) from regional weather stations rather than site-specific measurements taken at the exact time and location of agricultural work. In contrast, participants reported heat-related symptoms experienced during the previous 12 mo. This difference in temporal and spatial specificity may introduce exposure misclassification and limit the precision with which environmental heat estimates can be directly linked to individual health outcomes. Nevertheless, the WBGT estimates provide an important contextual indicator of environmental heat conditions in the region during peak agricultural months.

### Conclusion

This study underscores the pressing need to address occupational heat stress as a significant health risk among female agricultural workers in the Jordan Valley, Palestine. The findings highlight the link between heat-related health symptoms and inadequate workplace infrastructure, suggesting that policy interventions focusing on improving working conditions and ensuring access to essential resources could substantially reduce the health burden on these workers. Beyond documenting these risks, the study contributes to global debates on climate-linked occupational health by showing how heat stress is intensified through gendered labor precarity and political constraints, offering insights relevant to vulnerable agricultural workforces in similar contexts. These results highlight the importance of policy interventions that improve working conditions, ensure access to essential resources, and prioritize gender-sensitive labor protections. By addressing both the structural and physiological dimensions of heat exposure, the agricultural sector can better safeguard the health and well-being of its most vulnerable workers, enabling them to continue contributing to the agricultural economy without compromising their health.

## Supplementary Material

wxag048_Supplementary_Data

## Data Availability

The datasets used and/or analyzed during the current study are not publicly available in order to ensure participants' privacy and confidentiality, but are available from the corresponding author on reasonable request.
