# Supplementary material for: Occupational heat stress among female agricultural workers in the Jordan Valley, Palestine
Source: Ann Work Expo Health. 2026 Jun 22;70(5):wxag048. doi: 10.1093/annweh/wxag048 (PMC13285726; doi:10.1093/annweh/wxag048)
Supplement: wxag048_Supplementary_Data [file wxag048_supplementary_data.pdf]

# Supplementary Materials

# Occupational Heat Stress among Female Agricultural Workers in the Jordan Valley, Palestine

Authors:

**Maysaa Nemer[1], Suzan Mitwalli[1], Hanin Basha[1]**

[1] Institute of Community and Public Health, Birzeit University, Birzeit, Palestine

**Corresponding Author:**
Maysaa Nemer, MPhil, PhD
Associate Professor
Institute of Community and Public Health, Birzeit University
PO Box 14, Birzeit, West Bank, Palestine
Phone: +970-2-298-2020
Email: mnemer@birzeit.edu

**Supplementary Material 1: Sampling Methodology**

**Target population and sampling frame:**

The study targeted all agricultural working women aged 18 years and older, residing with their families in the Palestinian Jordan Valley in 2020.

According to the United Nations Office for the Coordination of Humanitarian Affairs (OCHA) classification, the Jordan Valley area includes 29 communities. (Additional communities exist but have very small populations.)

According to the Palestinian Central Bureau of Statistics (PCBS) 2017 Census, data are available for working women across both agricultural and non-agricultural sectors. Since the communities in this area are primarily agricultural (plant and animal production), it was assumed that at least half of the working women are engaged in agricultural activities.

The list of communities in the Jordan Valley is presented in Table 1, specifying the governorate and locality names, along with total population and working women data retrieved from the PCBS 2017 Census.

**Table 1: List of communities in Jordan Valley (2017)**

| # | Governorate | Locality name | Population "Census 2017" | Total working women, "Census 2017" |
|---|---|---|---|---|
| 1 | Tubas and Northern | Bardala | 1584 | 134 |
| 2 | Tubas and Northern | 'Ein el Beida | 1122 | 84 |
| 3 | Tubas and Northern | Kardala | 200 | 93 |
| 4 | Tubas and Northern | Ibziq | 127 | 9 |
| 5 | Tubas and Northern | Al Farisiya | 116 | 18 |
| 6 | Tubas and Northern | Al 'Aqaba | 166 | 4 |
| 7 | Tubas and Northern | Al Malih | 349 | 42 |
| 8 | Tubas and Northern | Khirbet ar Ras al Ahmar | 73 | 1 |
| 9 | Tubas and Northern | Khirbet 'Atuf | 213 | 7 |
| 10 | Tubas and Northern | Al Hadidiya | 180 | 4 |
| 11 | Tubas and Northern | Khirbet Yarzah | 31 | 0 |
| 12 | Tubas and Northern | Khirbet Tell el Himma | 76 | 8 |
| 13 | Nablus | 'Ein Shibli | 313 | 6 |
| 14 | Nablus | Furush Beit Dajan | 722 | 76 |
| 15 | Nablus | Alttawel and Tall al | 107 | 20 |
| 16 | Nablus | Biet Hasan | 1597 | 49 |

| | | | | |
|---|---|---|---|---|
| 17 | Nablus | An Nassariya | 1886 | 62 |
| 18 | Nablus | Al 'Aqrabaniya | 936 | 24 |
| 19 | Jericho & Al Aghwar | Marj Na'ja | 828 | 11 |
| 20 | Jericho & Al Aghwar | Az Zubeidat | 1679 | 179 |
| 21 | Jericho & Al Aghwar | Marj al Ghazal | 243 | 1 |
| 22 | Jericho & Al Aghwar | Al Jiftlik | 3099 | 79 |
| 23 | Jericho & Al Aghwar | Fasayil | 1636 | 10 |
| 24 | Jericho & Al Aghwar | Al 'Auja | 5204 | 124 |
| 25 | Jericho & Al Aghwar | An Nuwei'ma | 1793 | 110 |
| 26 | Jericho & Al Aghwar | 'Ein ad Duyuk al Fauqa | 884 | 35 |
| 27 | Jericho & Al Aghwar | Aqbat Jaber Camp | 8946 | 214 |
| 28 | Jericho & Al Aghwar | An Nabi Musa | 343 | 3 |
| 29 | Jericho & Al Aghwar | Jericho (Ariha) | 20690 | 1187 |
| **Total** | | | **55143** | **2594** |

**Sample size:**

The sample size was calculated using the standard formula for sample size determination described by William G. Cochran (Cochran 1977):

$$n = \frac{t^2 * s^2}{e^2} * Deff$$

When the total population is small, we can correct the sample size according to the following formula:

$$corrected\ \ n = \frac{n}{1 + \frac{N}{n}}$$

Where:

| | |
|---|---|
| **n** | Sample size |
| ***N*** | The total target population, it is supposed to be half of the total working women. |
| **t** | Is the factor that gives a level of confidence 95%, and it is equal to 1.96 |
| **$s^2$** | The variance of the main estimate in the study (which is supposed to be a proportion (p) and equal 50% to get the maximum sample size, where P=0.5, 1 –P = 0.5, $S^2$ = P (1-P) = 0.25 |
| **e** | Margin of error ≈ 0.05 on the total sample size |
| ***Deff*** | The design effect, because we use the two-stage cluster sample, we suppose the value of *Deff* = 1.2 |

After applying these calculations and adjustments, the final required sample size was 356 agricultural working women.

**Sample design:**

A two-stage stratified cluster sampling design was used:

1- First Stage: Selection of communities using Probability Proportional to Size (PPS) sampling across the three governorates. The size measure used for PPS selection was the total number of working women per community, based on data from the PCBS 2017 Census.
2- Second stage: Selection of agricultural working women within each selected locality.

**Strata:**

The main stratum is the governorates. So, the sample represented all three governorates that cover the Jordan valley area.

**Sample Allocation:**

The total sample was allocated proportionally across the three governorates, taking into account both the total population and the total number of working women in each governorate. This approach ensured adequate statistical representation of each governorate.

**Table 2: Sample of communities and of Agricultural working women**

| # | Governorate | Locality name | Sample size |
|---|---|---|---|
| 1 | Tubas and Northern Valleys | Bardala | 40 |
| 2 | Tubas and Northern Valleys | Kardala | 28 |
| 3 | Nablus | Furush Beit Dajan | 36 |
| 4 | Nablus | An Nassariya | 27 |
| 5 | Jericho & Al Aghwar | Az Zubeidat | 60 |
| 6 | Jericho & Al Aghwar | An Nuwei'ma | 50 |
| 7 | Jericho & Al Aghwar | Jericho (Ariha) | 115 |
| **Total** | | | **356** |

**Supplementary Material 2: Map of the study communities**

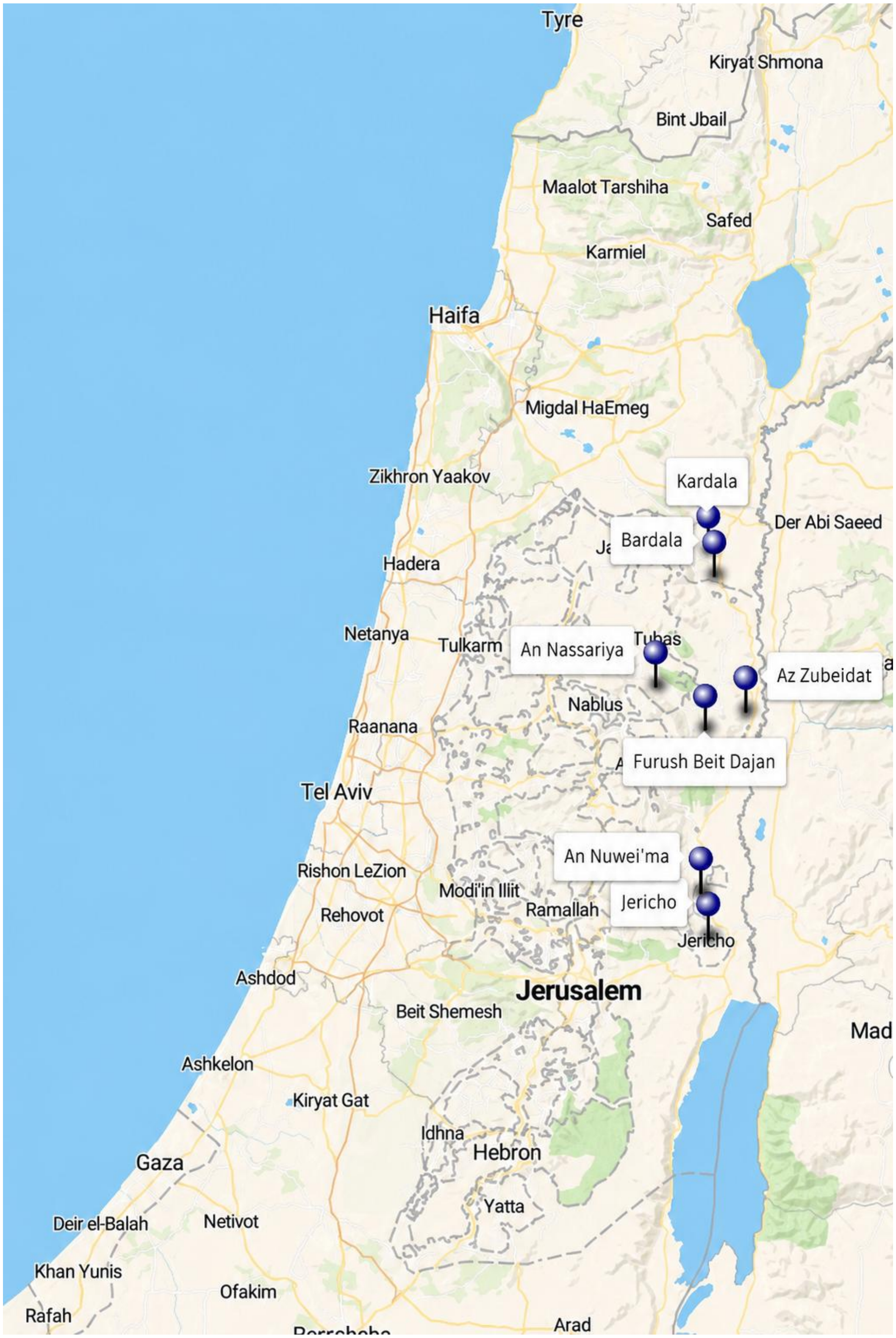

**Supplementary Material 3: Peak WBGT (°C) at the four time points in the three locations**

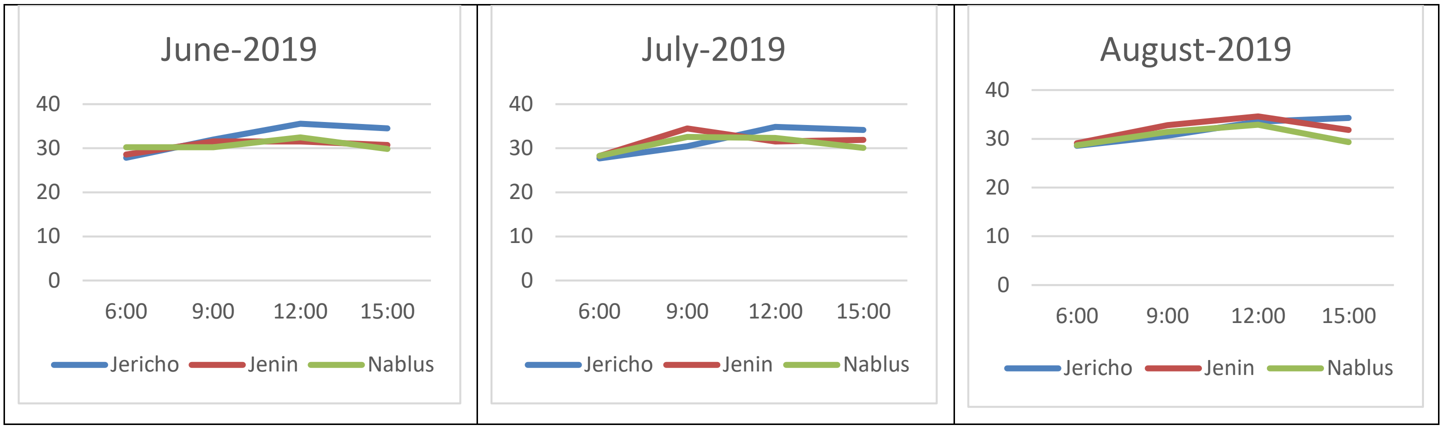

**Supplementary Material 4: Significant associations from bivariate analysis (Chi-square test)**

| | Excessive sweating (n=174) | Muscle/Heat cramps (n=133) | Excessive thirst (n=171) | Tiredness/ Weakness (n=308) | Dizziness (n=121) | Severe headache (n=206) | Kidney stones (n=36) | Pain in the lower back (n=252) |
|---|---|---|---|---|---|---|---|---|
| **Age group** | | | | | | | | |
| 41-50 | | | | | | * | | * |
| **Governorate** | | | | | | | | |
| Jericho | | | * | * | | | | |
| **Intensity of work** | | | | | | | | |
| Heavy | | | | | | * | * | |
| **Place of work** | | | | | | | | |
| Greenhouse | | | | ** | ** | ** | | ** |
| Field | | | | | ** | | | |
| **Vigorous work tasks** | | | | | | | | |
| Yes | ** | | ** | * | | | | |
| **An allocated place for eating, drinking, and resting** | | | | | | | | |
| No | | | | * | * | ** | | * |
| **Access to drinking water** | | | | | | | | |
| No | | | * | * | ** | ** | * | ** |
| **Access to a toilet** | | | | | | | | |
| No | | | | ** | ** | ** | * | ** |
| **Years of work** | | | | | | | | |
| 6-15 years | | * | | | * | | * | ** |
| More than 25 years | | | | | | * | | |
| **Months per year** | | | | | | | | |
| 5 months or less | | | | | ** | | | |
| 9-12 months | * | | * | | ** | | | |
| **Days per week** | | | | | | | | |
| 7 days | * | | * | ** | ** | ** | * | ** |

| | | | | | | | |
|---|---|---|---|---|---|---|---|
| **Hours per day** | | | | | | | |
| Less than 8 hours | * | * | ** | ** | ** | ** | ** |

*** P value < 0.05**
**** p value < 0.001**

## References:

Cochran WG. 1977. Sampling techniques third edition. New York: John Wiley & Sons.
